# Eosinophils prevent diet-induced airway hyperresponsiveness in mice on a high-fat diet

**DOI:** 10.1152/ajplung.00213.2024

**Published:** 2024-09-24

**Authors:** Becky J. Proskocil, Gina N. Bash, David B. Jacoby, Allison D. Fryer, Zhenying Nie

**Affiliations:** Division of Pulmonary and Critical Care Medicine, Oregon Health & Science University, Portland, Oregon, United States

**Keywords:** airway nerves, asthma, eosinophil, hyperinsulinemia, obesity

## Abstract

Eosinophils contribute to metabolic homeostasis and airway hyperresponsiveness, but their specific role in obesity-related airway hyperresponsiveness remains unclear. To address this, we used transgenic mice that overexpress interleukin-5 (IL-5) in peripheral T cells (+IL-5T) and wild-type controls. On a normal diet, +IL-5T and wild-type mice have similar body weight, body fat, and airway nerve-mediated reflex bronchoconstriction in response to inhaled serotonin. Feeding wild-type mice a 61.6% high-fat diet resulted in significantly increased body weight, body fat, fasting glucose, fasting insulin, and reflex bronchoconstriction induced by serotonin, which was blocked by vagotomy. In contrast, +IL-5T mice on a high-fat diet gained less body weight and fat than wild-type mice on the same diet and did not exhibit potentiation in fasting glucose, fasting insulin, or reflex bronchoconstriction induced by serotonin. Compared with wild-type mice, +IL-5T mice on normal diet had significantly more adipose tissue eosinophils, and this was further increased by high-fat diet. High-fat diet did not increase adipose tissue eosinophils in wild-type mice. Our findings suggest that adipose tissue eosinophils may play a role in regulating body fat, thereby reducing insulin, which is a mediator of obesity-related airway hyperresponsiveness. Thus, our data indicate adipose tissue eosinophils may be an important avenue for research in obesity-related asthma.

**NEW & NOTEWORTHY** This study investigates how eosinophils influence systemic metabolism and airway function in obesity. Known for their immune functions, eosinophils also mitigate obesity-related hyperinsulinemia, reducing airway hyperresponsiveness in obese mice models. The findings suggest potential therapeutic strategies targeting the intricate interplay among neurons, eosinophils, and the endocrine system to alleviate asthma in obesity. This research provides novel insights into the critical neuro-immune-endocrine interactions essential for managing obesity-related asthma.

## Graphical abstract

**Figure:**
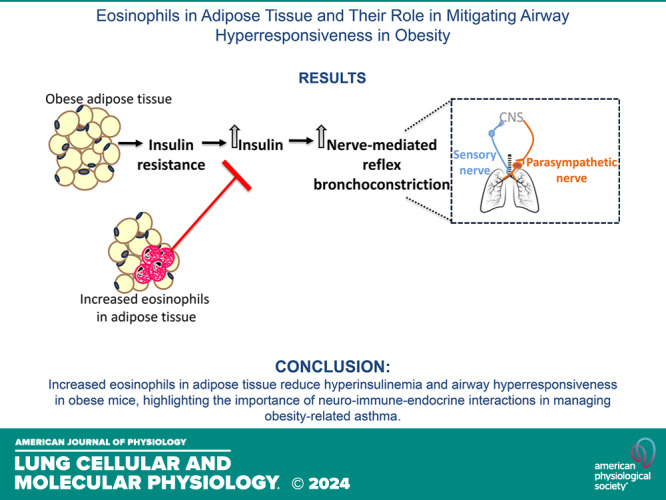

## INTRODUCTION

A major characteristic of asthma is airway hyperresponsiveness, which is mediated by dysfunctional airway nerves (1–4). In healthy lungs, airway nerves mediate bronchoconstriction via a reflex relayed by both airway sensory and parasympathetic nerves (2, 3). The dominant control of airway smooth muscle is provided by parasympathetic nerves that release acetylcholine onto M_3_ muscarinic receptors, resulting in smooth muscle contraction and airway narrowing (3, 4). Acetylcholine release is normally limited by inhibitory M_2_ muscarinic receptors on parasympathetic nerves (1, 2). Loss of M_2_ receptor function removes this negative feedback mechanism, significantly potentiating bronchoconstriction in animal models of asthma (2, 5, 6) and in patients with asthma (7, 8). Sensory nerves are distributed throughout the airway epithelium and send signals to the central nervous system that activate parasympathetic nerves to release acetylcholine onto M_3_ muscarinic receptors on airway smooth muscle. Increased density of sensory nerves in the lungs is also associated with airway hyperresponsiveness in animals and with severe asthma in humans (2). Reflex bronchoconstriction, induced by stimulating sensory nerves with an irritant or agonist, results in bronchoconstriction mediated by the parasympathetic nerves. This reflex can be potentiated either by increased sensory nerve activity or by increased acetylcholine release from parasympathetic nerves independently, or by an additive effect.

In obese animals, we linked metabolic dysfunction to nerve-mediated airway hyperresponsiveness (5, 6, 9–12). Rats and mice on a high-fat diet develop not only obesity but also hyperinsulinemia. Hyperinsulinemia acutely reduces neuronal M_2_ muscarinic receptor function, consequently increasing airway hyperresponsiveness to electrical stimulation of parasympathetic nerves (5, 10–12). Pharmacological depletion of insulin prevented the development of obesity-induced airway hyperresponsiveness (5, 10, 11). A role for sensory nerves has also been shown, as obesity-induced hyperinsulinemia increases the density of airway sensory nerve innervation (6). In addition, specific knockout of insulin receptors on sensory nerves (6) prevents the development of obesity-induced airway hyperresponsiveness.

Obesity-related asthma represents an asthma subtype that can be severe and is often difficult to treat (13–17). Eosinophils and other inflammatory cells are commonly associated with asthma, but obesity-related asthma does not respond to anti-inflammatory treatments (14, 16–18), suggesting a unique and novel mechanism, unrelated to increased inflammation. Outside the lung, eosinophils are present in normal adipose tissue in both mouse and humans (19, 20) and are increasingly recognized as regulators of adipose tissue metabolism and metabolic health (9, 21). There is a reduction in adipose tissue eosinophils in individuals with obesity (22). This negative correlation between adipose tissue eosinophils and weight gain has also been shown in rodents on a high-fat diet (19, 23). Importantly, in obesity, higher numbers of adipose tissue eosinophils are associated with decreased insulin resistance (19, 24–26), possibly by increasing insulin sensitivity (19, 24). Because we have shown that elevated insulin potentiates airway nerve-mediated bronchoconstriction in obese mice and rats, here, we tested whether increased adipose eosinophils inhibit airway nerve-mediated hyperresponsiveness in mice on a high-fat diet.

## METHODS

### Mice

ROSA26-EGFP [B6;129-Gt(ROSA)26Sor^tm2Sho^/J; #004077] mice and wild-type mice (C57BL/6J; #000664) were purchased from Jackson Laboratory (Bar Harbor, ME). Transgenic mice that overexpress interleukin-5 in T cells (+IL-5T) and have high levels of circulating eosinophils in the blood [NJ1638; (27)] and transgenic mice that express Cre recombinase under the eosinophil peroxidase promotor [eoCre; (28)] were gifted by Dr. Jamie Lee. All animals in this study were less than 7 mo of age and were healthy based on weight and visual observation by our laboratory staff and Oregon Health and Science University’s veterinarians. ROSA26-EGFP mice were crossed with eoCre mice to generate mice with green fluorescent protein (GFP)-expressing eosinophils (eoCre-GFP) and these mice were crossed with both the wild-type and +IL-5T mice to generate mice with GPF-labeled eosinophils. Mice were given ad lib access to food and water and were kept on a 12-h light/dark cycle. For most experiments, mice were fasted for 16 h before physiological and metabolic measurement to avoid fluctuations in blood insulin due to food consumption. For some experiments, measuring airway hyperresponsiveness following acute insulin (Fig. 5), mice were not fasted overnight. Animals were handled in accordance with protocols approved by the Institutional Animal Care and Use Committee at Oregon Health and Science University.

### Diet, Body Weight, and Body Fat

Adult male and female mice (5–6 wk old) were randomized to receive either a normal diet (13.6% fat, 28.9% protein, and 57.4% carbohydrate, LabDiet PicoLab 5L0D) or a high-fat diet (61.6% fat, 18.1% protein, and 20.3% carbohydrate, TestDiet 58Y1, St. Louis, MO) for 19 wk (6). Mice were weighed before they started the diet and after 19 wk of diet. Body fat was measured in fasted mice (16 h) using a nuclear magnetic resonance-based body composition analyzer (EchoMRI-5000, Houston, TX) as previously described (11). Percentage of body fat was calculated by dividing body fat by body weight. Food intake of high-fat diet was measured (each week) in grams, and daily calorie consumption per mouse was calculated based on the caloric content per gram of food.

### Airway Physiology

Airway physiology was measured in fasted (16 h) wild-type and +IL-5T mice after 19 wk of diet. We have previously reported no significant difference in airway nerve-mediated hyperresponsiveness between male and female mice on a high-fat diet (6). Thus, sex was not considered to be a variable in these experiments, and all data were combined from male and female mice. Mice were anesthetized with ketamine (100 mg/kg ip) and xylazine (10 mg/kg ip), tracheostomized for ventilation (120 breaths/min, 100% oxygen, 0.2-mL tidal volume) with a positive end-expiratory pressure of 2 cmH_2_O, and paralyzed with succinylcholine (20 mg/kg im) as previously described (29). Body temperature was monitored via a rectal probe and maintained at 36–37°C using a Homeothermic Monitoring System (Harvard Apparatus, Holliston, MA), and heart rate was monitored by electrocardiogram (Animal Bio Amp, ADInstruments, Colorado Springs, CO). Inspiratory flow was measured by a pneumotachograph (MLT1L) attached to a spirometer and change in airway pressure was measured by a pressure transducer (MLT0670, ADInstruments). A nebulizer (Aeroneb, Kent Scientific, Torrington, CT) was attached to the tracheal cannula for the nebulization of agents into the airways. Data were acquired by the PowerLab 8/35 and recorded using LabChart 8.1.19 software (ADInstruments).

Airway resistance was measured as previously described (29). Briefly, baseline pressure was restored by performing two deep inhalations at 25 cmH_2_O before nebulization with either PBS (10 μL) or increasing doses of serotonin (10–300 mM; 10 μL). Inspiratory holds were performed for 225 ms at peak pressure for six consecutive breaths. For each of those six breaths, end-inflation pressure (plateau pressure) during the inspiratory holds and peak pressures were recorded. Airway resistance was calculated as the difference between peak pressure and plateau pressure, divided by airway flow. Inspiration holds were conducted before and after each dose of nebulized serotonin to establish baseline and postdose measurements, respectively. The airway resistance for baseline and postdose was determined by averaging the measurements from the six breaths. The change in airway resistance induced by each dose of serotonin was calculated by subtracting the baseline airway resistance from the airway resistance measured after each dose. In some animals, a bilateral vagotomy was performed between a repeated series of inhaled serotonin to determine the neuronal contribution to airway resistance in response to serotonin. Some wild-type mice on normal diet received intraperitoneal human recombinant insulin (1.29 U/kg; Humalog; Eli Lilly, Indianapolis, IN) or PBS 30 min before measuring the increase in airway resistance in response to inhaled PBS and serotonin before and after bilateral vagotomy.

### Flow Cytometry

Blood and PBS-perfused lung and perigonadal adipose were collected from wild-type eoCre-GFP and +IL-5T eoCre-GFP mice after 19 wk on a normal diet or high-fat diet. Erythrocytes in the blood sample were lysed with water. Lung and adipose were cut into small pieces and digested in 2 mg/mL collagenase IV (17104019; Fisher Scientific, Waltham, MA) in RPMI 1640 media (Gibco, Grand Island, NY) at 37°C for 1–2 h in a shaking water bath. Digested tissue was processed through a mesh strainer and digestion was stopped with 3% fetal bovine serum (Cytiva HyClone, Fisher Scientific) in RPMI 1640 media. Cells were centrifuged at 500 *g* for 5 min at RT, resuspended in RPMI media containing 3% fetal bovine serum, and counted on a hemocytometer. Cells were centrifuged at 500 *g* for 5 min and resuspended in 1% bovine serum albumin (BP1600; Thermo Fisher Scientific; 30 min) to block nonspecific binding. Cells were incubated with CD45 antibody (APC-Cy™7 conjugated; 557659 BD PharMingen, San Diego, CA) in 1% bovine serum albumin for 1 h at RT. After the cells were washed, cells were fixed with 4% formaldehyde at RT for 15–30 min. Cells were analyzed for CD45 (all leukocytes) and GFP (eosinophils) on a FACSymphony flow cytometer (BD PharMingen) using FlowJo software (Tree Star, Ashland, OR). The number of eosinophils expressing GFP was counted and normalized to the number of leukocytes (CD45+ cells) in the tissue.

### Glucose and Insulin Measurements

Blood was collected from fasted (16 h) wild-type and +IL-5T mice after fed a normal diet or high-fat diet for 19 wk. Blood glucose was measured on a OneTouch Ultra 2 (LifeScan, Inc, Malvern, PA) using blood collected from the tail vein. Minutes later, blood from the inferior vena cava was collected to measure serum insulin by a mouse insulin ELISA (10–1247-01, Mercodia, Uppsala, Sweden). Glucose and insulin measurements were performed in samples collected from the same animal.

### Statistical Analysis

The number of mice used in this study was determined both by previous experience with similar studies and using a power analysis. Statistical analysis of body weight after diet, body fat, flow cytometry, fasting glucose, and fasting insulin data were performed using two-way ANOVA and post hoc Sidak test. Body weight before diet and caloric intake data were compared using two-tailed Student’s *t* test. To reduce subjective bias, physiological data were blinded before analysis. Due to the visual difference in size between mice on a high-fat diet and those on a normal diet, it was not feasible to collect physiological data in a fully blinded manner. To reduce bias during processing and statistical analysis, only animal identification numbers (excluding treatment or genotype information) were recorded on LabChart recordings, tissue samples, blood samples, and metabolic measurements. Thus, the analysis of physiological data was conducted blind. Airway physiology pre- and postvagotomy data were analyzed using two-way repeated-measures ANOVA and post hoc Sidak test. Data were analyzed using GraphPad Prism 10.0.1 (La Jolla, CA) and expressed as mean and standard deviation (SD). Significance is indicated as *P* ≤ 0.05.

## RESULTS

### +IL-5T Mice on High-Fat Diet Gain Less Weight and Less Body Fat

**Figure 1.**
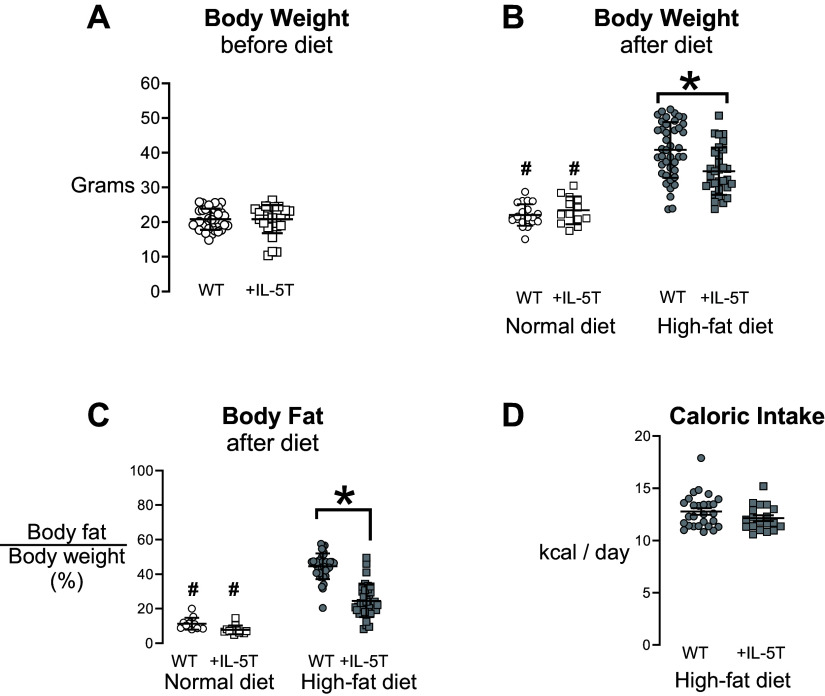
+IL-5T mice gain less weight and less body fat on a high-fat diet than wild-type mice. Wild-type (WT; circles) and +IL-5T mice (squares) were fed either normal diet (open symbols) or high-fat diet (filled symbols) for 19 wk. Body weight was recorded before starting the diet (*A*). After 19 wk of normal or high-fat diet, body weight (*B*) and body fat (*C*) were measured. Caloric intake was calculated for wild-type and +IL-5T mice on a high-fat diet (*D*). Individual mice are graphed and displayed with the means and SD. *n* = 13–42 for each group. A Student's *t* test was performed to compare body weight before diet and caloric intake. A two-way ANOVA with a post hoc Sidak test was used to compare body weight and body fat after diet. #*P* < 0.05 indicates a significant difference in body weight (*B*) and body fat (*C*) for both genotypes on the normal diet compared to the high-fat diet. *P ≤ 0.05 indicates a significant difference between +IL-5T and wild-type mice on the high-fat diet.

Wild-type and +IL-5T mice had similar weight before starting the treatment diet (Fig. 1*A*). After 19 wk of normal diet or high-fat diet, both wild-type and +IL-5T mice fed a high-fat diet significantly gained body weight (Fig. 1*B*) and body fat (Fig. 1*C*) compared with respective mice on a normal diet. However, +IL-5T mice on a high-fat diet (filled squares) experienced significantly less body weight gain (Fig. 1*B*) and accrued less additional body fat (Fig. 1*C*) than wild-type mice fed a high-fat diet (filled circles). Importantly, this reduction in body weight gain and body fat accumulation was not due to reduced caloric intake (Fig. 1*D*).

### High-Fat Diet Induces Airway Hyperresponsiveness in Wild-Type but Not +IL-5T Mice

**Figure 2.**
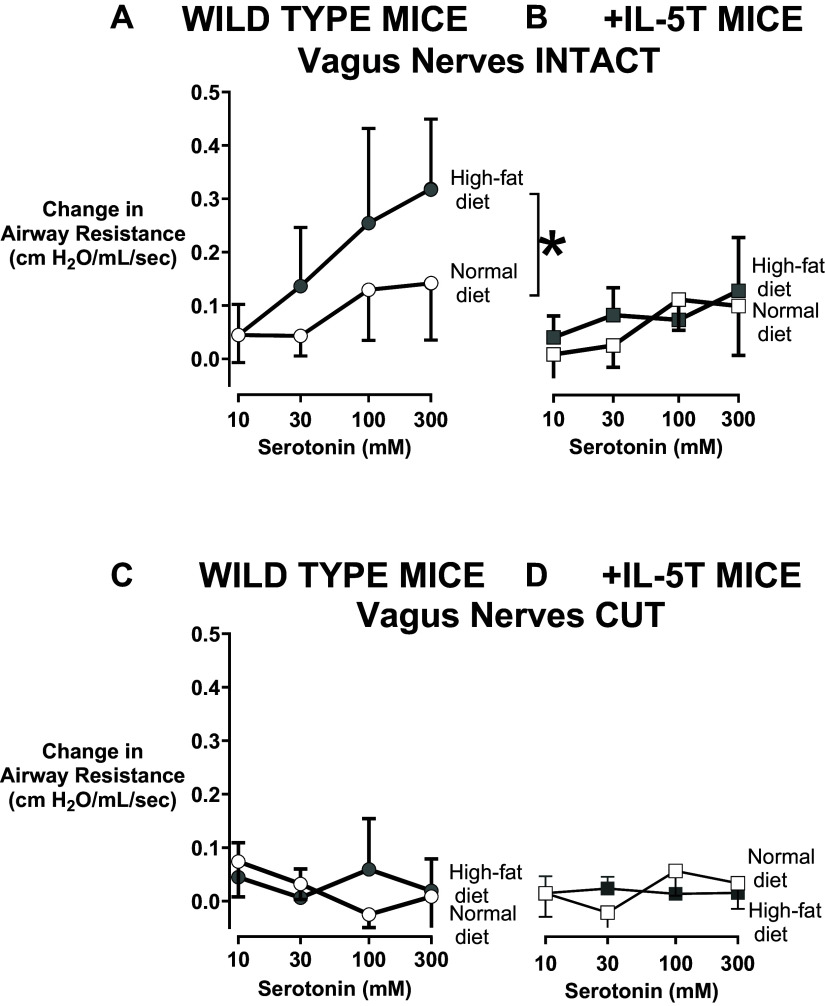
High-fat diet induces nerve-mediated airway hyperresponsiveness in wild-type (WT) but not +IL-5T mice. Change in airway resistance was measured in response to increasing doses of inhaled serotonin (10–300 mM) in +IL-5T mice (squares) or wild-type mice (WT; circles) fed a high-fat diet (filled symbols) or normal diet (open symbols) for 19 wk. Changes in airway resistance in response to increasing doses of serotonin were potentiated in wild-type animals on a high-fat diet before (*A*) but not after (*C*) vagotomy showing that airway hyperresponsiveness requires an intact nervous system. High-fat diet did not potentiate airway hyperresponsiveness in +IL-5T mice either before or after vagotomy (*B* and *D*). Data are graphed means and SD for each group. *n* = 5–12 for each group. Data were analyzed by a two-way repeated-measures ANOVA and post hoc Sidak test. *P ≤ 0.05.

Wild-type mice on a normal diet showed a dose-dependent increase in airway resistance in response to inhaled serotonin (Fig. 2*A*; open circles), which was significantly potentiated in wild-type mice fed a high-fat diet (filled circles). Similarly, serotonin also induced a dose-dependent increase in airway resistance in +IL-5T mice fed a normal diet (Fig. 2*B*; open squares) that was not statistically different from wild-type mice fed a normal diet (Fig. 2*A*; open circles). In contrast to wild-type mice, high-fat diet did not potentiate airway resistance in +IL-5T mice (Fig. 2*B*). Increased airway resistance in response to inhaled serotonin in all animals requires an intact nervous system as all serotonin-induced bronchoconstriction was abolished by bilateral vagotomy (Fig. 2, *C* and *D*). In the absence of serotonin, there was no difference in baseline airway resistance among wild-type mice on a normal diet (mean = 0.84 cmH_2_O/mL/s, SD = 0.23), wild-type mice on a high-fat diet (mean = 0.85 cmH_2_O/mL/s, SD = 0.09), +IL-5T mice on a normal diet (mean = 0.76 cmH_2_O/mL/s, SD = 0.12), and +IL-5T mice on a high-fat diet (mean = 0.80 cmH_2_O/mL/s, SD = 0.08).

### High-Fat Diet Increases Eosinophils in Adipose Tissue of +IL-5T Mice

**Figure 3.**
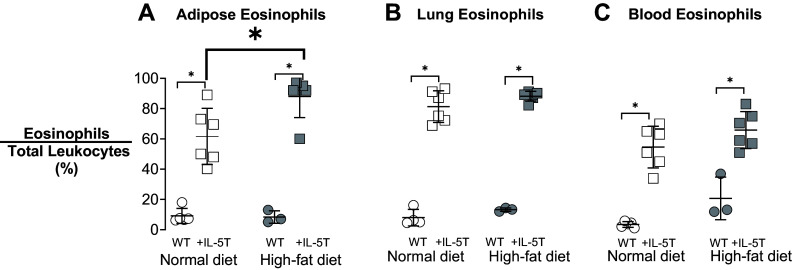
High-fat diet increases eosinophils in adipose tissue of +IL-5T mice. eoCre-GFP/wild-type (WT; circles) mice and eoCre-GFP/+IL-5T (squares) mice were fed normal diet (open symbols) or a high-fat diet (filled symbols) for 19 wk. After 19 wk, adipose tissue (*A*), lung tissue (*B*), and venous blood (*C*), were collected, digested, and labeled with antibody to CD45 to measure total leukocytes. Eosinophils were identified by positive GFP expression. Using flow cytometry, leukocytes (CD45+), and eosinophils (GFP+) were sorted and counted. Eosinophils were significantly increased in all +IL-5T mice, compared with wild-type, in all three compartments. After 19 wk on a high-fat diet, there was an additional, significant increase in adipose tissue eosinophils only in +IL-5T mice (*A*). The percentage of eosinophils in relation to total leukocytes was calculated and compared within each tissue. Individual mice are graphed and displayed with the means and SD. *n* = 3–6 for each group. Data were analyzed with a two-way ANOVA and post hoc Sidak test. *P ≤ 0.05.

Eosinophils, tagged with GFP (28), were collected from adipose, lung, and blood from wild-type and +IL-5T animals. +IL-5T mice (squares), regardless of diet, had significantly more eosinophils in adipose (Fig. 3*A*), lung (Fig. 3*B*), and blood (Fig. 3*C*) than wild-type mice (circles). High-fat diet did not alter the number of eosinophils in blood, adipose tissue, or lung in wild-type mice. In contrast, the number of eosinophils in adipose tissue from +IL-5T mice fed a high-fat diet (filled squares) was significantly increased compared with +IL-5T fed a normal diet (open squares) (Fig. 3*A*), while the number of lung (Fig. 3*B*) and blood (Fig. 3*C*) eosinophils remained the same.

### Fasting Blood Glucose and Insulin Were Decreased in +IL-5T Fed a High-Fat Diet

**Figure 4.**
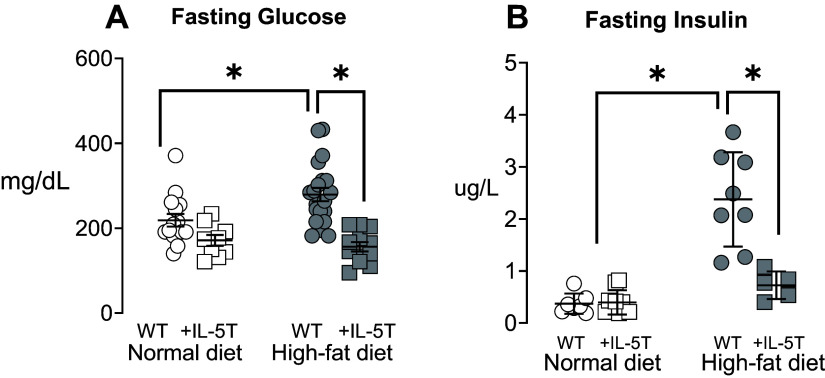
+IL-5T mice (squares) had decreased fasting blood glucose (*A*) and blood insulin (*B*) compared with wild-type mice (WT; circles) after being fed a high-fat diet (filled symbols) for 19 wk. Normal diets are shown in open symbols. Individual mice are graphed and displayed with the means and SD. *n* = 4–12 for each group. Data were analyzed by a two-way ANOVA and post hoc Sidak test. *P ≤ 0.05.

Baseline glucose in wild-type mice on a normal diet (mean = 175 mg/dL, SD = 24) is comparable to data published by Jackson Laboratory for this strain (30). On a normal diet, fasting blood glucose was slightly decreased in +IL-5T mice compared with wild-type mice (Fig. 4*A*), but fasting insulin remained the same (Fig. 4*B*). In wild-type mice fed a high-fat diet, fasting glucose and insulin were significantly increased (Fig. 4, *A* and *B*). Fasting glucose and insulin were not increased in +IL-5T mice fed a high-fat diet (Fig. 4, *A* and *B*).

### Insulin Administered Intraperitoneally Increases Airway Resistance

**Figure 5.**
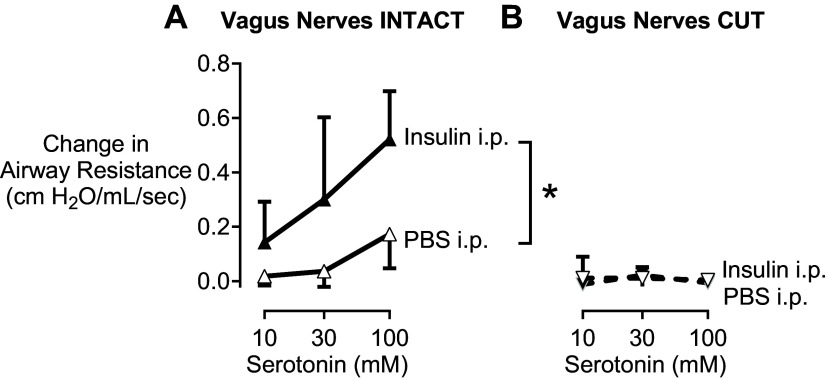
Acute intraperitoneal administration of insulin (filled triangles), but not PBS (open triangles) induces airway hyperresponsiveness in wild-type mice fed a normal diet. Increases in airway resistance in response to increasing doses of inhaled serotonin (10–100 mM) were significantly potentiated in animals before (*A*) but not after (*B*) vagotomy showing that airway hyperresponsiveness to acute insulin requires an intact nervous system. Data are represented as means and SD. *n* = 7 for each group. Data were analyzed by a two-way repeated-measures ANOVA and post hoc Sidak test. *P ≤ 0.05.

To confirm the role of insulin on modulating airway hyperresponsiveness, anesthetized and ventilated wild-type mice on a normal diet were administered 1.29 U/kg recombinant human insulin intraperitoneally. Thirty minutes later, airway resistance in response to inhaled serotonin was measured. Acute administration of insulin (filled triangles) significantly increased airway resistance compared with wild-type mice that received PBS intraperitoneally (open triangles; Fig. 5*A*). Following bilateral vagotomy, bronchoconstriction responses to inhaled serotonin were abolished, indicating serotonin-induced bronchoconstriction was neuronally mediated (Fig. 5*B*). These mice were not fasted to avoid hypoglycemia with acute insulin treatment. There was no significant difference in serotonin responsiveness between fasting (Fig. 2*A*; wild-type normal diet) and nonfasting mice (Fig. 5*A*; PBS ip). Neither was there any difference in baseline resistance in wild-type mice that were fasted (mean = 0.84 cmH_2_O/mL/s, SD = 0.23) and wild-type mice that were not-fasted (mean = 0.90 cmH_2_O/mL/s, SD = 0.14) before serotonin administration (*P* = 0.65).

## DISCUSSION

Airway nerve-mediated hyperresponsiveness is the crucial mechanism underlying obesity-related asthma. Hyperinsulinemia, a consequence of obesity, exacerbates airway nerve-mediated bronchoconstriction (5, 6, 10, 11). Here, we show a novel protective role of adipose tissue eosinophils in transgenic mice with increased systemic eosinophils, inhibiting obesity-related airway hyperresponsiveness. Our data are consistent with existing literature suggesting that adipose tissue eosinophils can reduce obesity (24) and high insulin (31) in mice on high-fat diets. Importantly, our data demonstrated that increased adipose tissue eosinophils prevented airway nerve-mediated reflex hyperresponsiveness in obese mice, by inhibiting hyperinsulinemia. Our study provides novel insights into the critical neuro-immune-endocrine interactions essential for managing obesity-related asthma.

High-fat diets potentiate nerve-mediated reflexes that increase airway responsiveness in rats and mice (5, 6, 10, 11). It is worth noting that high-fat diet did not increase bronchoconstriction mediated by contraction of airway smooth muscle. Our data show that cutting the vagus nerves, thereby removing the neural contribution to serotonin-induced bronchoconstriction, eliminated all airway hyperresponsiveness. This finding is consistent with our previous research in guinea pigs (32), rats (5), sheep (33), and mice (29), which indicates that airway smooth muscle responsiveness to muscarinic agonists or serotonin is not potentiated in vagotomized animals, even after they have been virus-infected, antigen challenged, exposed to ozone, treated with organophosphorus pesticides, or fed a high-fat diet.

High-fat diets also increase circulating insulin in rats and mice and insulin potentiates nerve-mediated airway hyperresponsiveness (5, 6, 10, 11). In rats on a normal diet, acute administration of insulin intraperitoneally potentiated bronchoconstriction in response to electrical stimulation of the vagus nerve (5). Here, we similarly show that insulin immediately potentiated serotonin-induced reflex bronchoconstriction in wild-type mice on a normal diet (Fig. 5). Insulin has been shown to reduce neuronal M_2_ muscarinic receptor expression on parasympathetic nerves (34) and acutely inhibits M_2_ muscarinic receptor function (5). Notably, neuronal M_2_ muscarinic receptor dysfunction is a well-recognized contributor to asthma pathogenesis in both human and animal models (8, 35–37). Insulin also promotes sensory nerve growth (38) and increases sensory innervation of airway epithelium (6), which is another mechanism resulting in increased reflex-induced bronchoconstriction. Reducing circulating insulin with metformin (10) or pioglitazone (11) prevents obesity-related airway hyperresponsiveness in rats. +IL-5T mice, with increased circulating eosinophils, on high-fat diet had low levels of fasting insulin and did not have airway hyperresponsiveness. Thus, insulin emerges as a crucial factor driving airway hyperresponsiveness in individuals with obesity.

Separate from insulin, airway hyperresponsiveness in asthma is often associated with increased eosinophils. When eosinophils are activated within the airway, they can have detrimental effects on lung tissue and airway physiology (39). Approximately 39–45% (40, 41) of asthma is eosinophilic, and acute exacerbations are controlled with therapeutics (42) that target IL-5, a cytokine required for eosinophil maturation and survival. Eosinophils can modulate both parasympathetic and sensory nerves in asthma. Eosinophils are actively recruited to airway nerves, where they release preformed cationic proteins, including eosinophil major basic protein, which is an allosteric antagonist of M_2_ muscarinic receptors (43). Eosinophils are found next to parasympathetic nerves and ganglia in lungs of antigen-challenged guinea pigs and humans with fatal asthma (44). Depletion of eosinophils (45, 46), blockade of eosinophil migration to the lung (45), and blockade or removal of eosinophil major basic protein (47) protect or restore neuronal M_2_ muscarinic receptor function and prevent or reverse airway hyperresponsiveness. Eosinophils are also associated with sensory nerves in airway epithelium (48), where there is a significant association between eosinophils and increased sensory nerve density and increased asthma severity (48). Increased sensory nerve density results in increased parasympathetic activation via the central reflex. However, it is noteworthy that the presence of eosinophils in lungs is not sufficient to induce airway hyperresponsiveness. Eosinophils need to be increased around airway nerves, in close proximity to parasympathetic nerves (44, 49) and also activated to release major basic protein (46, 49–51).

Here, we tested the role of eosinophils in obesity-related airway hyperresponsiveness that we know to be mediated by insulin and airway nerves. +IL-5T mice at baseline have increased circulating eosinophils, including in the lung, but airway nerve-mediated reflex bronchoconstriction was not potentiated in response to inhaled serotonin (Fig. 2*C*) likely because eosinophils in +IL-5T mice are not concentrated around airway nerves (29), thereby exerting no effect on nerve-mediated airway bronchoconstriction. Although wild-type mice on a high-fat diet developed increased insulin and airway hyperresponsiveness, +IL-5T mice fed a high-fat diet did not develop either increased insulin or airway hyperresponsiveness.

Eosinophils exhibit diverse biological functions influenced by their specific microenvironment (26). In adipose tissue, eosinophils collaborate with macrophage type 2 cytokines to promote transformation of white adipocytes into beige adipocytes (52, 53), increasing glucose uptake, fatty acid oxidation, and lipolysis, thereby indirectly enhancing insulin sensitivity (54). This contributes to energy expenditure, preventing obesity (55). An inverse correlation of adipose tissue eosinophils with body weight and glucose sensitivity has been previously reported (19, 31). Our data show that +IL-5T mice on a high-fat diet had a significant increase in adipose tissue eosinophils and gained less weight than wild-type mice. Increased adipose tissue eosinophils in +IL-5T mice may contribute to enhanced energy expenditure. Likewise, +IL-5T mice on a high-fat diet had significantly lower blood glucose and insulin than wild-type mice. This effect was not due to increased blood eosinophils, as +IL-5T mice fed normal diet also had significantly increased blood eosinophils compared with wild-type mice but did not have decreased fasting glucose or insulin compared with the wild-type mice.

The mechanism behind increased eosinophils in adipose tissue is unknown. In humans, obesity-related asthma is not consistently associated with increased circulating eosinophils (17, 56, 57). The mechanism behind increased eosinophils in adipose tissue in +IL-5T mice is unknown. B-cells are also increased in the IL-5 transgenic mice used here (27). However, increased B-cells alone are unlikely to contribute to airway hyperresponsiveness as depleting eosinophils in delta double GATA mice (24) increased B-cells but did not induce airway hyperresponsiveness (58), disassociating increased systemic B-cells from airway hyperresponsiveness. Eotaxin 1 is an important cytokine for eosinophil recruitment and is expressed by adipocytes. However, van den Berg et al. (59) showed that high-fat diet decreased mRNA and protein for eotaxin 1 in adipose tissue. Therefore, it seems unlikely that eotaxin 1 alone is responsible for the increased eosinophil presence in the adipose tissue of +IL-5T mice. Thus, pharmacological targets to manipulate adipose eosinophils that might benefit obesity-related asthma are unknown.

The physiological role of eosinophils is complicated and is a highly active area of research. New data are emerging that propose different eosinophil phenotypes and subgroups, based on microenvironment, atopic status, and cell age. Here, we show a beneficial effect of eosinophils in adipose tissue reduces circulating insulin and reverses nerve-mediated airway hyperresponsiveness in obese mice. Although the association between adipose eosinophils and reduced insulin resistance has been demonstrated in previous studies (19, 24, 25, 31, 60), our findings highlight the downstream effects, particularly the reduction of circulating insulin and its impact on airway function. Our research provides novel insights into the critical neuro-immune-endocrine interactions that are essential for managing obesity-related asthma. Better understanding of mechanisms describing how adipose eosinophils modulate circulating insulin, adipose inflammation, and airway hyperresponsiveness is a novel and promising avenue for research in obesity and obesity-related asthma.

Mice on a high-fat diet develop obesity, insulin resistance, and compensatory hyperinsulinemia, which can enhance airway nerve-mediated bronchoconstriction. Our study reveals that increased eosinophils in adipose tissue reduce hyperinsulinemia and specifically mitigate airway hyperresponsiveness in an obese mouse model. These findings highlight potential therapeutic strategies targeting the interplay among neurons, eosinophils, and the endocrine system to alleviate obesity-related asthma. This research provides new insights into the crucial neuro-immune-endocrine interactions involved in managing this condition.

## DATA AVAILABILITY

Data will be made available upon reasonable request.

## GRANTS

Financial support was provided by the National Institutes of Health – National Heart, Lung, and Blood Institute (NHLBI) Grants HL163087 (to Z.N. and A.D.F.), HL164474 (to Z.N.), NIH S10OD034444 (to Z.N.), HL144088 (to D.B.J.), HL131525 (to A.D.F.), and F30HL154526 (to G.N.B.); and National Institute of Allergy and Infectious Disease (NIAID) Grant AI152498 (to D.B.J.).

## DISCLOSURES

No conflicts of interest, financial or otherwise, are declared by the authors.

## AUTHOR CONTRIBUTIONS

D.B.J., A.D.F., and Z.N. conceived and designed research; B.J.P., G.N.B., and Z.N. performed experiments; B.J.P., G.N.B., and Z.N. analyzed data; B.J.P., G.N.B., D.B.J., A.D.F., and Z.N. interpreted results of experiments; B.J.P., G.N.B., and Z.N. prepared figures; B.J.P. and Z.N. drafted manuscript; B.J.P., G.N.B., D.B.J., A.D.F., and Z.N. edited and revised manuscript; B.J.P., G.N.B., D.B.J., A.D.F., and Z.N. approved final version of manuscript.
